# Intestinal intussusception of Meckel’s diverticulum, a case report and literature review of the last five years

**DOI:** 10.31744/einstein_journal/2023RC0173

**Published:** 2023-01-27

**Authors:** Dora Sandoval Schaedlich, Pedro Custodio de Mello Borges, Arnaldo Lacombe, Renato Alonso Moron

**Affiliations:** 1 Faculdade Israelita de Ciências da Saúde Albert Einstein Hospital Israelita Albert Einstein São Paulo SP Brazil Faculdade Israelita de Ciências da Saúde Albert Einstein, Hospital Israelita Albert Einstein, São Paulo, SP, Brazil.; 2 Hospital Israelita Albert Einstein São Paulo SP Brazil Hospital Israelita Albert Einstein, São Paulo, SP, Brazil.

**Keywords:** Meckel diverticulum, Intestine, small, Diverticulum, Intussusception, Laparoscopy, Ileal diseases

## Abstract

Meckel’s diverticulum is the most common gastrointestinal tract anomaly. It arises from the incomplete closure of the omphalomesenteric conduit, which is a true diverticulum at the antimesenteric border of the ileum. Although the majority of patients are asymptomatic, they can present with inflammation, hemorrhage, intussusception, intestinal obstruction, and perforation, among others; this constitutes an important differential diagnosis for acute abdomen. A 19-year-old female sought medical attention because of intermittent diffuse abdominal pain for two months, nausea, and diarrhea. In the requested imaging tests, tomography, and enterotomography, a diagnosis of Meckel’s diverticulum with some degree of intussusception was suggested. The patient underwent elective surgical treatment without complications and was discharged on the second postoperative day with clinical improvement. In this section, we review publications on similar cases published in the last five years.

## INTRODUCTION

Meckel’s diverticulum (MD) is the most common gastrointestinal tract anomaly with an estimated prevalence of 2% in the population.^(1,2)^ Its pathophysiological process consists of an incomplete closure of the omphalomesenteric (vitelline) conduit, forming a true diverticulum at the antimesenteric border of the small intestine. Most cases are asymptomatic, and the discovery of the diverticulum requires examinations for other causes during the surgical approach. However, sometimes there may be clinical symptoms; for example, with inflammation generating a picture of diverticulitis, a differential diagnosis of an acute inflammatory abdomen would be suggested. The diagnosis of MD is usually made by the association of clinical suspicion with imaging tests, which may not have satisfactory sensitivity and specificity, sometimes requiring a surgical and anatomopathological approach to establish the diagnosis with certainty. Within the MD, ectopic mucosa can be found, the most frequent being gastric and pancreatic mucosa, but the presence of these findings is not mandatory. Thus, the clinical picture of MD may present with ulceration and bleeding in the gastrointestinal tract due to ectopic gastric mucosa. Meckel’s diverticulum can cause other complications, such as hemorrhage, intussusception, intestinal obstruction, perforation, and, very rarely, bladder diverticular fistula and tumors. Treatment usually consists of surgical resection of the diverticulum.^(3)^

## CASE REPORT

A 19-year-old female patient sought emergency care due to a complaint of diffuse abdominal pain, which was greater in the lower abdomen, associated with mild nausea and diarrhea. She had a history of intermittent chronic pain and had been in medical care at this service for two months prior for the same reason, with abdominal ultrasound and laboratory tests within the limits of normality. New ultrasound and laboratory tests were requested, in addition to prescribing analgesic medications.

**Figure 1:**
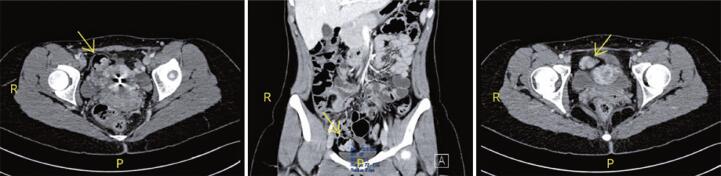
Examination images - computed tomography, portal phase, performed as an emergency, without oral contrast. Yellow arrows point to a blind-end loop in the right iliac fossa, raising a diagnostic hypothesis of Meckel’s diverticulum

The ultrasound result again showed no abnormalities, and emergency computed tomography (CT) was performed as shown in figure 1; in the venous phase, a blind-end loop in the right iliac fossa that could correspond to MD, with emphasis on parietal thickening and casting of the same, with contrast medium enhancement. No other significant changes were observed. Complementing the study with oral contrast was suggested to help better characterize the findings.

**Figure 2:**
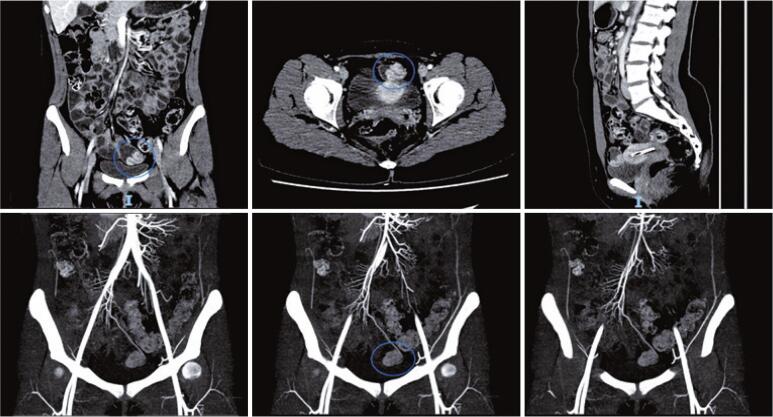
Examination images - enterotomography performed with negative oral contrast and with arterial phase for vascular evaluation. Blue circles demonstrate the presence of a diverticulum, which is mobile compared to the previous study, in this one in the left paramedian region of the pelvis with mucosal hyper-enhancement and suggestion of invagination of the same. Feeding artery is observed along the diverticulum

The investigation continued with enterotomography and CT with oral and venous contrast to better assess the hypothesis of MD in the arterial phase and vascular study, which was performed two days later. The findings in figure 2 confirm the presence of MD, mobile in the comparison between the tomographic studies, located in the median/left paramedian region of the pelvis, superior to the bladder dome. She had significant mucosal thickening and hyperenhancement of her pons, with an image suggesting partial invagination of the pons. A nourishing artery was highlighted along the diverticulum and inserted at the tip. Peridiverticular adipose planes with preserved attenuation can be seen. The remaining thin loops had a normal distribution, without significant parietal thickening. No fistulous pathways, organized collections, or lymph node enlargements were observed.

The patient remained clinically stable, with no changes in laboratory test results and no clinical signs of obstruction or other acute intestinal complications. Elective surgery was scheduled 19 days after the CT scan.

**Figure 3:**
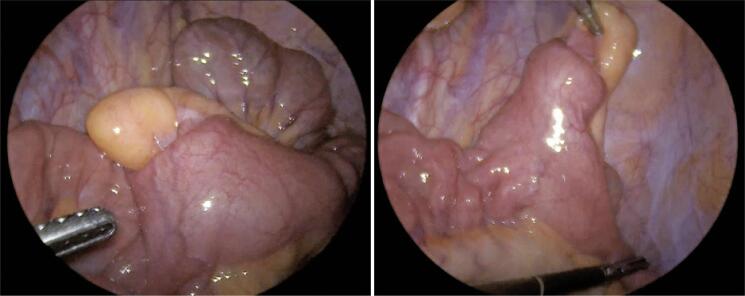
Screen photo in videolaparoscopy. Left, Meckel’s diverticulum invaginated into the lumen of the small intestine; on the right, Meckel’s diverticulum rectified out of the small intestine lumen

The surgical procedure was videolaparoscopy, performed with the patient under general anesthesia. A Meckel’s diverticulum with a wide base and partial invagination was identified (Figure 3). Segmental enterectomy with resection of the diverticulum and intracorporeal mechanical side-to-side anastomosis is the treatment of choice. The procedure continued with closure of the mesenteric gap, review of hemostasis, removal of the surgical piece, and closure of the portals and dressings. The procedure was uneventful.

**Figure 4:**
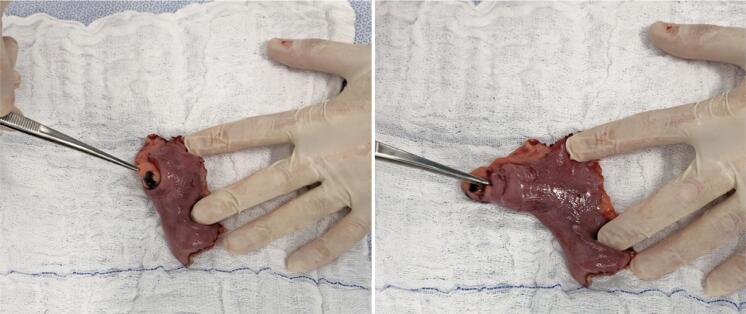
Photo of the specimen resulting from intestinal resection. On the left, a photo of a piece containing an invaginated Meckel’s diverticulum; on the right, photo of the part containing the rectified Meckel’s diverticulum

Macroscopically figure 4, the material resulting from the resection was described as a segment of small intestine measuring 7.0cm in length and 3.0cm in perimeter, which presented a smooth and shiny serosa, with a saccular area measuring 2,5cm x 2.3cm and 2.5cm from the end.

The anatomopathological report was compatible with MD, described as a saccular projection of the enteric wall lined by the gastric fundic epithelium with foveolar hyperplasia. The remaining enteric wall had a well preserved architecture. Viable surgical margins and no morphological evidence of malignancy were noted.

The patient evolved well clinically and was discharged on the second postoperative day. This study was approved by the Research Ethics Committee of *Hospital Israelita Albert Einstein* under CAAE: 59733422.0.0000.0071; #5.548.250.

## DISCUSSION

We performed a search in the PubMed database on February 8, 2022, looking for publications that contained the descriptors (intussusception) and (Meckel) or (Invagination) and (Meckel) in their titles to obtain articles similar to the present report. The search yielded 178 items, and the timeline of publications was quite broad. The first publication dates from 1902, being a case of an adult American patient with an acute obstructive abdomen due to the invagination of MD. Interestingly, the author cites a similar presentation, described by a colleague in 1884.^(4)^The latest date is 2022.

**Figure 5:**
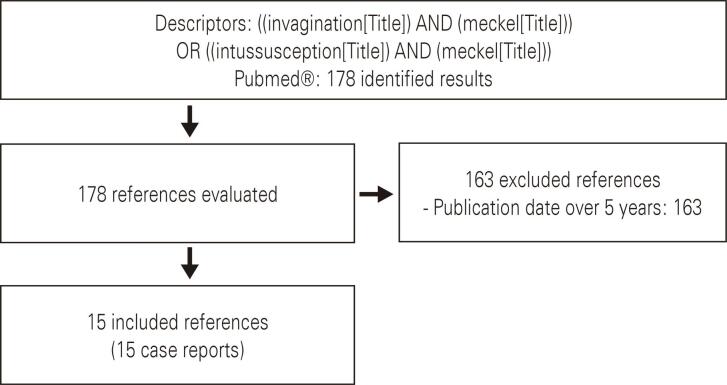
Flowchart for selection of studies

We decided to revisit the reports of the last 5 years in this discussion, therefore determining as exclusion criteria any article with a publication date beyond this period. This represents 15 publications, all of which are represented by case reports in figure 5.

### Epidemiology

From a geographical perspective, we observed that 15 publications came from five continents. One was from America (United States),^(5)^ four from Europe (Italy,^(6)^ France,^(7)^ Spain^(8)^ and the United Kingdom),^(9)^ one from Africa (Tunisia),^(10)^ seven from Asia (Japan,^(11-14)^ South Korea,^(15)^ China^(16)^ and Syria)^(17)^ and two from Oceania (Australia).^(18,19)^

Regarding the age of the patients included in this sample, there was a range from newborns to 49 years of age. Although in the literature, children up to 10 years of age represent more than 50% of symptomatic MD cases,^(3)^ the incidence in the reports reviewed in this discussion proved to be slightly different. Two patients were less than 6 months of age,^(6,10)^ three between 1 and 6 years of age,^(14,16,17)^ five between 15 and 30 years of age^(5,7,13,18,19)^and five between 40 and 50 years of age.^(8,9,11,12,15)^

Seven males and eight females were included in the study. However, the tendency for symptomatic presentations of MD is more prevalent in males, with a ratio ranging from 1.5:1 to 4:1.^(3)^

### Symptoms and presentation by age

Of the patients younger than 6 years old, the most prevalent symptom was vomiting (60%), followed by abdominal pain (40%), hematochezia (40%), and lowered level of consciousness (40%). One patient was initially investigated for shaken baby syndrome, constipation (20%), and fever (20%). On physical examination, 40% had no changes, 20% had abdominal distension, 20% had abdominal stiffness, 20% had abdominal pain, and 20%, (that is, one case), had lower limb edema, and was diagnosed with protein-losing enteropathy in addition to intestinal intussusception with necrosis due to MD.

In the database, among children with symptoms due to MD, 46.7% had obstruction, 25.3% had gastrointestinal tract bleeding, and 19.5% had inflammation.^(3)^

In the category of patients over 15 years of age, the most commonly described symptoms were abdominal pain in 90% of cases, nausea and/or vomiting in 60% of cases, bowel movement arrest and flatus in 10%, hematochezia in 10%, and asymptomatic in 10% (that is, a patient with no complaints who was diagnosed by an accidental finding in the investigation of another etiology). Regarding physical examination, 20% of the reports did not comment on this evaluation, while in the others, there was a 50% prevalence of pain on abdominal palpation, 25% abdominal distension, 25% pain on decompression, and 38% no changes.

From the available data on symptomatic MD in adults, we found that 35.6% had obstruction, 27.3% had gastrointestinal tract hemorrhage, and 29.4% had inflammation.^(3)^

### Complementary exams

Of the studies cited in this article, at least one imaging test was requested for all cases. Laboratory findings of leukocytosis were described in 2 of 15 reports. The most prevalent tomographic findings were intestinal loop distention and diagnosis of intussusception, followed by free fluid in the cavity, lesion with a target appearance, tumor progression in size, edematous thickening, and cystic lesions. The most cited sonographic finding was the “target sign,” followed by other descriptions of intussusception, a report of double intussusception, a suspected volvulus, and an image with free fluid in the cavity.

As we have seen above, the symptoms and pathological processes that cause them are not specific to MD, which is a diagnostic challenge due to the possibility of considering other etiologies of acute abdomen. Thus, diagnostic complementation with imaging examinations or depending on the case of surgical complementation for investigation of the condition is of paramount importance. Some tools commonly cited for follow-up are radiography, ultrasound, tomography, magnetic resonance imaging, angiography, arteriography, and nuclear scans with CT-99m pertechnetate.^(3)^

### Surgical findings

Regarding the intraoperative findings, of the 15 cases, we observed that in 46% of them, there was a description of necrosis/ischemia, and of these, one report of perforation. In 33% of the cases, there was diagnostic doubt due to the description only of the detection of an intraluminal lesion/tumor, and the remaining 66% had classic intussusception resulting from MD. One patient even had double intussusception. Primary anastomosis was performed in all surgeries.

Once the diagnostic hypothesis of MD has been raised, or another etiology of surgical treatment has been proposed as a differential diagnosis, direct observation of MD will provide the correct diagnosis. This can be performed surgically, by laparoscopy or laparotomy, or even with endoscopy of the small intestine or capsule endoscopy; importantly, each method has its indications and reservations regarding specificity and sensitivity. However, it is important to reiterate, as mentioned above, that it is not uncommon for asymptomatic cases or cases under investigation/approach to other clinical situations in which MD is accidentally found, and its resection at diagnosis has been advocated by some authors.^(3)^

### Anatomopathological

All reports confirmed the diagnostic hypothesis of MD. In addition, 26.6% of patients had ectopic pancreatic tissue, 20% had ectopic gastric tissue, and 6.6% had ectopic gastric and pancreatic tissue. One report did not describe the anatomopathological findings.

However, there has been a description that the presence of gastric ectopia is the most common tissue found, being present in 4.6% to 71% of symptomatic MD, followed by pancreatic tissue in 0% to 12%. These two factors are responsible for 97% of the ectopias present in MD; however, duodenal or colonic tissue may also have been present.^(3)^

### Outcomes

In all cases described, good surgical recovery was observed, and the patients were discharged with clinical improvement. A need for surgical re-approach was described in one patient for lysis of adhesions 12 months after discharge.

## CONCLUSION

We describe a case report of a patient with Meckel’s diverticulum and intestinal intussusception secondary to invagination of the diverticulum, with a clinical presentation and surgical and anatomopathological findings consistent with most cases published in the last five years. Considering that Meckel’s diverticulum is a prevalent intestinal malformation, this etiology should be considered as an important differential diagnosis when faced with abdominal complaints.

## References

[1] Sagar J, Kumar V, Shah DK (2006). Meckel’s diverticulum: a systematic review. J R Soc Med.

[2] Elsayes KM, Menias CO, Harvin HJ, Francis IR (2007). Imaging manifestations of Meckel’s diverticulum. AJR Am J Roentgenol.

[3] Hansen CC, Søreide K (2018). Systematic review of epidemiology, presentation, and management of Meckel’s diverticulum in the 21st century. Medicine (Baltimore).

[4] Wainwright JM (1902). IV. Intussusception of Meckel’s diverticulum. Ann Surg.

[5] Zorn J, Zhang S, Brandt J, Keckeisen G (2022). Small bowel obstruction precipitated by intussusception of Meckel’s diverticulum. SAGE Open Med Case Rep.

[6] Guanà R, Pagliara C, Zambaiti E, Scottoni F, Pane A, Garofalo S (2021). Incidental Ultrasound Diagnosis of Neonatal Intussusception Secondary to Meckel’s Diverticulum in a Neurologically Impaired Child. Am J Case Rep.

[7] Sendy F, D’escrivan T, Joubert A, Fetche N (2020). Double intussusception secondary to Meckel’s diverticulum in a seventeen-year-old female: a case report. Pan Afr Med J.

[8] Martínez Mojarro R, González Benjumea P, García Del Pino B, Perea Sánchez MJ (2019). Recurrent abdominal pain due to intussusception of Meckel diverticulum. Cir Esp (Engl Ed).

[9] McGrath AK, Suliman F, Thin N, Rohatgi A (2019). Adult intussusception associated with mesenteric Meckel’s diverticulum and antimesenteric ileal polyp. BMJ Case Rep.

[10] Louati H, Zouari M, Jallouli M, Dhaou MB, Zitouni H, Mhiri R (2017). Perforated Meckel’s diverticulum causing intussusception in a neonate. J Neonatal Surg.

[11] Yamauchi N, Ito T, Matsuoka H, Chohno T, Hasegawa H, Kakeji Y (2020). Intussusception caused by a small intestinal lipoma with ectopic gastric mucosa containing gastric cystica profunda component cells within the inverted Meckel’s diverticulum: a case report. Surg Case Rep.

[12] Abe I, Saito M, Ikeda T, Fukuda R, Tanaka A, Rikiyama T (2020). Ileectomy performed on a case of adult intussusception due to inversion of Meckel’s diverticulum. J Surg Case Rep.

[13] Kawasaki Y, Shinozaki S, Yano T, Oshiro K, Morimoto M, Kawarai Lefor A (2017). Intussusception due to an Inverted Meckel’s Diverticulum Diagnosed by Double-Balloon Enteroscopy. Case Rep Gastroenterol.

[14] Tei E, Hirakawa H, Mori M, Hirabayashi T, Ueno S (2017). Protein-losing enteropathy caused by spontaneous reduction of intussusception with Meckel’s diverticulum. Tokai J Exp Clin Med.

[15] Kang SI, Gu MJ (2020). Synchronous ileal inflammatory fibroid polyp and Meckel’s diverticulum found during laparoscopic surgery for adult intussusception. Yeungnam Univ J Med.

[16] Yu M, Fang Z, Shen J, Zhu X, Wang D, Shi Y (2018). Double simultaneous intussusception caused by Meckel’s diverticulum and intestinal duplication in a child. J Int Med Res.

[17] Badour M, Hammed A, Baqla S (2021). Lethargy as an initial symptom of intussusception secondary to Meckel’s diverticulum in a 2.5 year-old girl: case report. Ann Med Surg (Lond).

[18] Dickfos M, Nabi H (2021). Inverted Meckel’s diverticulum: a rare cause of adult intussusception. ANZ J Surg.

[19] Combes AD, Limmer AM, Verschuer K (2020). Small bowel intussusception secondary to Meckel’s diverticulum containing polypoid lesion in pregnancy. ANZ J Surg.

